# Practical and Ethical Aspects of Advance Research Directives for Research on Healthy Aging: German and Israeli Professionals’ Perspectives

**DOI:** 10.3389/fmed.2018.00081

**Published:** 2018-04-05

**Authors:** Perla Werner, Silke Schicktanz

**Affiliations:** ^1^Department of Community Mental Health, University of Haifa, Haifa, Israel; ^2^Institute of Medical Ethics and History of Medicine, University Medical Center Göttingen, Göttingen, Germany

**Keywords:** advance research directives, dementia, professionals’ opinion focus group, healthy aging research, ethics, Germany, Israel

## Abstract

**Background:**

*Healthy aging* is the development and maintenance of optimal cognitive, social and physical well-being, and function in older adults. Preventing or minimizing disease is one of the main ways of achieving healthy aging. Dementia is one of the most prevalent and life-changing diseases of old age. Thus, dementia prevention research is defined as one of the main priorities worldwide. However, conducting research with persons who lack the capacity to give consent is a major ethical issue.

**Objective:**

Our study attempts to explore if and how advance research directives (ARDs) may be used as a future tool to deal with the ethical and practical issues in dementia research.

**Method:**

We conducted focus groups and in-depth interviews with German and Israeli professional stakeholders from the fields of gerontology, ethics, medical law, psychiatry, neurology and policy advice (*n* = 16), and analyzed the main topics discussed regarding cross-national similarities and controversies within the groups, as well as across the two national contexts.

**Results:**

While both countries are in the midst of a developmental process and have recognized the importance and need for ARD as a tool for expanding healthy aging, Germany is in a more advanced stage than Israel because of the EU regulation process, which indicates the influence of international harmonization on these research-related ethical issues. Consensual themes within the qualitative material were identified: the need for a broader debate on ARD, the ethical importance of autonomy and risk–benefit assessment for ARD implementation, the role of the proxy and the need for the differentiation of types of dementia research. Controversies and dilemmas aroused around themes such as the current role of IRBs in each country, the need for limits, and how to guaranty safeguarding and control.

**Discussion:**

Implementing a new tool is a step-by-step procedure requiring a thorough understanding of the current state of knowledge as well as of the challenges and hurdles ahead. As long as improving quality of life and promoting autonomy continue to be core elements in the process of healthy aging, efforts to advance knowledge and solve dilemmas associated with the implementation of ARD is of the utmost importance.

## Introduction

As the population worldwide ages (1), the focus in gerontology and geriatrics is moving from the treatment and management of disease to the promotion of healthy aging. While multiple and sometimes controversial definitions have been used to describe healthy aging (2), all share several common attributes. First, healthy aging is conceptualized as a process occurring along the life course (3). Second, it includes a multi-dimensional approach encompassing the development and maintenance of optimal cognitive, social and physical well-being and function (4). Third, sustained independence and autonomy were defined as fundamental in the process of achieving and maintaining healthy aging (5, 6). Finally, increasing multidisciplinary research in the area of healthy aging has been defined as essential (3, 7). Advancing research and knowledge regarding healthy aging and dementia is of the utmost importance.

*Dementia*, defined as a syndrome of severe progressive cognitive deterioration that impairs daily functioning, is one of the most prevalent and life-changing diseases of old age (8). Increasing dementia prevention research has been defined by Alzheimer’s Disease International and the World Health Organization as one of the main priorities worldwide (9). Such research cannot focus only on healthy participants, but needs to involve persons with dementia—whether in a comparative setting or in order to test for the long-term effects of particular treatments. Furthermore, this research might include long-term and large cohort studies, which means that participants’ cognitive capacity might decline over time.

Recruiting and retaining people who lack the capacity to give consent has been defined as one of the main crises necessitating the advancement of research in the area of AD and dementia (10). Most importantly, this problem confronts researchers with serious ethical problems. While advance research directives (ARDs) might provide a potential tool to deal with these issues, knowledge in the area remains scant.

The aim of this article is to examine the literature on the topic of ARD, with a special focus on the participation of persons with decreased cognitive capacity, and to explore the attitudes and perceptions of professional stakeholders about the topic in Israel and Germany. The chapter opens with a review of available knowledge in the area of ARD, followed by a description of the findings of expert interviews (in a focus group or as individuals) conducted in Israel and Germany in 2017. We will conclude the chapter by discussing the findings of our focus groups and their relevance regarding international efforts to introduce ARD as a tool for expanding research with persons with deteriorated cognitive functioning.

### ARD––A Brief Overview

The international ethics guidelines regarding research with persons lacking decisional competence is not very homogenous (11). In most countries, current research with persons with dementia relies—if at all allowed—on surrogate decision-making as “proxy consent.” This is the decision made by a formal, legally appointed guardian, a power of attorney or sometimes an informal representative (e.g., a family member consenting to a specific study).

However, this surrogate decision-making has lately been criticized for two reasons: first, it only allows for research with “minimal risk,” or for research with personal or patient group benefits—therefore, any research beyond minimal risk or for third parties cannot be conducted (12, 13). Second, surrogate decision-making is not fully in accordance with the ethical principle of patient self-determination—a principle which is increasingly gaining priority in international medical law and ethics.

Advance research directives might provide a potential way to overcome these criticisms. However, although the topic of ARD has been frequently discussed since the end of the previous century, clear legal regulations are still lacking in most European countries as well as in the United States (14–16). In the next subsections, we summarize what is known in the ARD literature till today. We limit our examination to ARD, but will refer to Advanced Health Care Directives when it was mentioned by the participants.

#### Defining the Concept

Advance research directives are legal documents allowing persons who have decisional capacity to express their preferences regarding participation in future research studies in the event they will lack this capacity to do so at the time of the research (12, 14). ARD differ from the concept of *advanced informed consent* because they document the individual’s interest and desire regarding potential future research, in general, rather than specific studies (17).

Appointing a specific proxy (also described as power of attorney) can also be part of an ARD. The recognition and implementation of ARD are lately being encouraged as a formal strategy to complement surrogate decision-making (12, 18, 19), and as a mechanism to increase respect for autonomy and the exercise of self-determined decision-making (20).

#### The Ethical Basis of ARD

Similar to Advanced Health Care Directives (AHD), the core ethical principles mentioned in the literature as the basis for ARD are self-determination, autonomy and empowerment (12, 14, 16, 19, 21, 22). The main understanding is that a person should determine by his/herself, in advance, what should be decided on his/her behalf in case he/she loses the capacity to make decisions (20). However, a common objection to this idea is that the will of the person might not remain the same in the current research situation, compared with when he/she decided and documented his/her will in the past. However, at this point he/she will no longer be able to express this change in attitude (23). Therefore, the idea of ARD serving “self-determination” in the current situation might be misleading. However, if we perceive *autonomy* as being relational, processual, and as self-expression through the support and interpretation of others, then ARD/AHD may be contextualized as justified instruments of autonomy (24, 25). Indeed, Jongsma and van de Vanthorst (20) discuss the dilemma between respect for autonomy and the "best interest" principle embedded in the ARD concept, and advocate for perceiving ARD as a morally defensible and reasonable basis for including persons with dementia, who lack the capacity to make decisions precisely because, in their view, respecting autonomy also means respecting preferences regarding their own future. Both claim further that research is different than care, as in the case of research, the anticipation of one’s best interest is less evident than perhaps in the case of receiving the best standard of general health care. As Jongsma (26) maintains, thus, the ARD and proxy consent must not be perceived as excluding alternatives, but as a way of providing more evidence for future proxies about how to make decisions in concrete situations as well as guide the proxy about how to ensure the ARD is respected by the different professionals in charge.

#### Safeguards for ARD Implementation

The ethical principle of respecting autonomy in research is normally implemented in practice as “informed consent.” In addition, an ARD should be based on informed consent in the sense that providing expanded, adequate information and education about the meaning of ARD as well as about different research scenarios and risk potentials is mandatory (19, 23). However, the overall aim of ARD implementation is twofold: protecting the subjects and ensuring self-determination on the one hand, while fostering research participation on the other. Therefore, it remains unclear whether ARD will actually increase or decrease research practice, and this might rely on how the research needs, risks and benefits are presented. Surrogates also need clear guidelines regarding their role, rights and duties when interpreting and acting on behalf of an ARD (12). Finally, close and steady contact and the monitoring of participants’ well-being are mentioned as the main safeguards to be respected by researchers and professionals (20, 23).

#### Prevalence Rates and Correlates of ARD

While information about this topic is scant and relatively outdated, studies examining these issues consistently show low prevalence rates (18, 21, 27, 28), and three main correlates of ARD: previous research experience, health care directives and the level of risk or side effects involved in the research protocol. Finally, it should be noted that there is no knowledge at all regarding these issues either in Germany or in Israel.

In sum, ARD is convincingly suggested as a new ethical-legal tool to discuss and ensure more self-determined wishes regarding research participation. However, many practical and ethical issues remain unclear or unsolved regarding the “what?” (“What needs to be described in an ARD? For which type of research is ARD needed?”); the “when?” (“When is the best time to convey information to others and encourage the public or patients to compose an ARD?”); the “who?” (“Who or which group of persons should be approached for an ARD?”); and the “how?” (“How should ARD be implemented in practice and which kind of safeguards are needed?”).

In the next section, we describe tentative responses to these questions as discussed in stakeholders’ interviews conducted in Israel and Germany on the topic.

### Comparing Professional Stakeholders’ Perspectives in Israel and Germany

Israel and Germany provide an ideal basis for comparison, since they are both characterized by public health care systems in which the topic of dementia has gained particular attention over the last years. However, neither country has developed a concrete dementia action plan or a particular policy regarding research on dementia or on healthy aging at all. In both countries, legal requirements regarding research with persons with dementia are rather restrictive. The latest change in Germany occurred in 2016 when, according to a new EU clinical trial regulation group, it was decided that research should be allowed with cognitively impaired persons, even the research only benefits this class of patients, but not the patient him/herself (hereafter labeled as “patient group benefit”). The political compromise ended by allowing such research only on behalf of the existence of an ARD, without any public or more detailed expert debate defining the pros and cons for such an ARD (29, 30). In Israel, a National Strategic Plan to Address Alzheimer’s and Other Types of Dementia was formulated in 2013 (31). While AHD are included in this plan as one of the main areas needing further development and awareness, the topic of ARD is not directly mentioned. Furthermore, advisory committees regarding dementia were established in both countries: In Germany, the so-called Alliance for People with Dementia has, since 2012, provided a platform to inform the Ministry of Health and the Federal Ministry for Family Affairs, Senior Citizens, Women and Youth with ideas and information related to dementia care. In Israel, the National Council for Dementia, established in 2013, focuses on improving training and research in the field of dementia.

## Materials and Methods

In order to explore the practical status of ARD and related ethical issues from a professional stakeholders’ perspective, we conducted two expert focus groups and additionally, two individual expert interviews (because several professionals were not available for the time scheduled for the focus groups) in Israel and Germany between March and September 2017. We use the term “expert” in a broad sense, as this includes scientific experts from a particular field (neurology, clinical geriatrics or social gerontology, bioethics, legal studies), as well as representatives of patient organizations or persons with practical expertise (e.g., on decision-making processes in ethics or policy committees). The experts we invited are also seen as professional stakeholders in the sense that they present legitimate interests and concerns of their field or their academic organization into the broader public or health policy debate (direct *via* policy advice or indirect by publications or presentations, newspaper comments, etc.). Focus groups were chosen as the method of inquiry because they create a shared space for group discussion in addition to allowing participants to expand the scope of the topic (32).

### Participants

A purposive sampling technique was used. A total of 16 participants from different fields (ethics, medicine, medical law, gerontology, dementia research, patient representation and health politics/insurance) participated. Seven experts took part in Germany: four experts had a background in medical ethics/medical law; one in clinical dementia care and research; one from gerontology; and one representative of a patient organization, who was also part of a ministerial board for dementia (gender ratio: four women, three men). In Israel, overall 9 professional stakeholders took part: three from medical ethics/medical law; two from clinical dementia care or research practice; three from gerontology; and one from ministerial administration (gender ratio: seven women and two men). The experts’ work experience ranged from 4 to 40 years.

Participants were promised anonymity for publication to allow a free and open-minded discussion.

### Procedure

Participants were recruited through the researchers’ professional networks while ensuring the experts’ well-known status of expertise by their documented research/working profile. We also considered the various disciplinary backgrounds for reaching heterogeneity. Focus group discussions were held until saturation of new information was reached (32). Before each focus group, all participants were asked to complete a short questionnaire, including demographic and professional information. Focus group meetings lasted on average almost 120 min. Discussions were audio taped and transcribed. The facilitator (SS) in both cases was skilled and experienced in conducting focus groups. In Israel, the meetings were conducted in English; in Germany, they were conducted in German. The main parts of the German transcript were translated into English for further comparative content analysis. For the purpose of publication, all original quotes are anonymized and only the professional background is mentioned. Our comparative qualitative content analysis was supported by using the scientific software ATLAS.Ti^®^ and was guided by the aim to first find similar topics and perspectives. The second step involved searching for cross-national specificities or professional peculiarities.

### Interview Guide

According to the recommended focus group methodology (32), a semi-structured interview guide containing open questions was developed by the research team (see Supplementary Material). The aim of the interview guide was to cover the following key themes: (a) professional experience and background knowledge of ARD; (b) assessing content and practical implementation of ARD; (c) overall perspectives on advanced planning in health issues; (d) assessment of the current dementia research setting and legal status in the respective country. The guide was developed jointly in English and afterward translated into German for the focus group in Germany. For the two additional individual expert interviews, we used the similar semi-structured interview guide.

The moderator also made sure to show enough flexibility to allow for open discussions among the participants.

## Results

The main topics emerging from the discussions are summarized in Tables 1 and 2. Table 1 provides an overview of topics and opinions that were shared by the majority or consensually discussed in the focus groups and showed similarities across German and Israeli stakeholders. These consensual topics can be categorized into the following main areas: Concept and need of ARD, ethical issues such as autonomy and risk–benefit assessment, the role of proxy, and desirable ARD content.

**Table 1:** Overview of main topics consensually discussed with the majorities’ opinion in the German and Israeli focus groups.

Main areas discussed	Consensual opinions
Concept and need of ARD	The need of advance research directives (ARD) must be further discussed and explored within the public and the professional community. Research in dementia and healthy aging studies would benefit from the increased participation of persons with dementia. Patients’ interest in taking part in research is high. The demarcation between ARD and AHD needs to be clarified: ARD might be a practical subpart of AHD, but as they cover research where the patient does not always benefit personally, there is a risk of therapeutic misconception or misuse. It is ineffective to approach the general public to sign ARD; instead, persons in early stages of dementia/Mild-cognitive impairment or if there is genetic disposition for a kind of dementia should be approached.
Ethical issues: Autonomy	ARD is a good tool for empowering patients and allowing them to express their own wishes regarding research participation, but demented persons remain a vulnerable population. Competency and capacities to compose an ARD are needed: any layperson might need a lot of information about the potential research and limitations of ARD. Approaching potential candidates for ARD needs to be done with sensibility and caution. ARD is not similar to consent; If an ARD states the wish to take part in research, it does still not imply a professional duty to include the patient in research ARD resamples AHD if the wish not to take in research is stated as this is a veto right for any research participation
Ethical issues: risk–benefit assessment	IRBs still have the main responsibility to assess the risks and benefits of a particular research study; ARD cannot replace the continuous monitoring and safeguarding of the patient’s best interests and actual opinions/desires. Misuse in research needs to be identified and avoided (responsibility of IRBs and researchers). Conflict of interest (research/career vs. care for and protection of patients) remain problematic, even when an ARD exists. Training of professionals and the IRB are crucial to implementing ARD properly, including monitoring the use and interpretation of ARD during a study. Undue burden must in all cases be avoided
Role of proxy	The role of proxy remains very important as a safeguard; in regard to concrete decisions, the proxy needs to balance the patient’s welfare and his/her future wishes.
ARD Content, type of research	Differentiation between various types of studies is needed and the public must be educated about these differences (e.g., what differences exist among observational studies, invasive vs. intervention studies; longitudinal epidemiological studies, cohort studies, etc.)

**Table 2:** Overview of main controversial topics discussed in German and Israeli focus groups.

Controversial issues/dissent	Israel	Germany
Current IRB/research practices	Disagreement as to whether non-invasive, observational studies are allowed under the current Israeli law (the heterogeneous practice might be due to the local IRB’s interpretation of what a risk or benefit actually entails)	Disagreement about whether non-invasive, observational studies are allowed under the old German law (the heterogeneous practice might be due to the local IRB’s interpretation of what a minimal risk or benefit actually entails) Disagreement about whether a once-given informed consent is still valid in longitudinal studies, during which the research participants gradually become demented, according to the current law.
Need of ARD	Controversy about whether a power of attorney is sufficient or even a better tool than advance research directives (ARD). Controversy about whether an ARD should also allow research that has neither a personal nor a/patient group benefit, but would be only benefit the public good.	Controversy about whether neither ARD nor a proxy should be an ethico-legal condition to allow research with persons with dementia if the research lacks any personal benefit Minority opinion that there is an ethical “slippery slope” in broadening the patient group benefit criteria to include any third party benefit in the future
Ethical Issues	Lack of clarity regarding concrete rights and responsibilities of *de facto* legal or informal guardians, family proxies or a power of attorney: who is best for a person with dementia? Uncertainty and doubts about how to monitor the well-being of research participants with dementia: neither an IRB nor a power of attorney have the skills to fulfill this type of monitoring	Concerns about lay persons’ competency to decide about research issues
Future ARD practice	Uncertainty over whether forms or pre-formulated texts are needed and what the patients preferences might be Disagreement about whether the low public motivation to compose an ARD/AHD can be explained by people’s tendency to deny death, aging and dementia or by the Israeli cultural attitude to put high trust in family for informal care.	Uncertainty about to whom and how information should be provided to patients/potential research candidates. Concern that there are problems in the interpretation of ARD, similar to AHD: the documents do not comply with clinical complexity and people change their minds during the course of a disease.

Overall, professionals in both groups recognized the need to find a mechanism to allow increased research activity involving persons with dementia who have diminished decisional capacity. In both countries, the need stemmed from the increasing individual and social costs faced by health systems because of the world’s ongoing demographic changes. Furthermore, it was also argued that the current restrictions on research with persons with dementia deprive this group of patients of evidence-based treatments. Disseminating and expanding knowledge about ARD among clinicians and the public was discussed in both groups and most agreed there was a need for more information and for conducting open discussions on the topic. Stakeholders in both countries extensively discussed implementation issues. The main common topic in this area referred to the connection between AHD and ARD and how research needs more safeguards and monitoring than treatment.

Participants conceptualized ARD as part of the process of respecting a person’s decision-making preferences and autonomy regarding research, but only in relation to his/her general wishes, and not, for example, in regard to veto rights regarding withdrawing/withholding treatment at the end of life. ARD were seen by most as a tool—if correctly done and based on proper information—to respect patients’ autonomy and personal wishes. However, proper risk–benefit assessment was seen as an important safety measure, in cases of persons with dementia who were seen as a vulnerable group. The majority agreed that research without personal benefit, but rather the benefit of the group, should only be conducted on minimal risk/minimal burden level. However, the notion of whether or not ARD might also allow for more than minimal risk/burden proved to be very controversial (see below). During the discussion about ARD, other common themes of research ethics were also mentioned. These themes mainly included IRBs and researcher’s responsibility. These basic principles were perceived as indicative of allowing research and monitoring during the study to determine whether any signs of burdening or objection might occur with the patient, independent of whether an ARD is available or not. The majority of participants in both countries also agreed that clear definitions of what is minimal risk/minimal burden and how exactly personal benefit vs. patient group benefit is determined is, in many cases, not easy: standards for defining these important issues do not exist. This also resulted in sharing the observation that there are heterogeneous decision policies in local IRBs in both countries regarding how restrictive or permissive research is assessed (see below and in Table 2).

Despite the different levels of knowledge and use found between both countries, it should be noted that the discussion about safeguard mechanisms for the use of ARD was extensive and far-reaching in both countries. Developing a monitoring system to follow the individual progress and status of each participant during the research project was one of the main concerns in both countries. Here, IRBs, researchers and legally appointed guardians were mentioned as the ones who “ensure” the well-being of research participants with dementia. Similarly, being aware of the different requirements for ARD depending on the research type (clinical, non-clinical) and the level of risk (no side effects, serious side effects; invasive, non-invasive) was a central topic in both countries. There was an overall consensus that it would be a good idea to inform patients and laypersons about the different types of research, their general risks and benefits, and to support the composing of such ARDs with forms/guidelines.

Despite these similarities, considerable differences and/or disagreements emerged in the focus groups regarding knowledge and attitudes. Table 2 provides an overview of the main topics that were rather controversially discussed by the stakeholders in both countries. These included: whether there is a real need for ARD; how ARD should be practically implemented; ethical issues such as the role of the proxy; what autonomous decisions means, and whether monitoring and safeguarding will work; and how the current practice in IRBs about dementia research or similar cases takes place.

Interestingly, the level of knowledge and familiarity of the professionals in both countries with the concept of ARD varied. While in Israel many participants mentioned that they had been exposed to the term (not the concept) for the first time during the focus group, professionals in the German study were well acquainted with it. The reason for the latter is the current legal change (see above) in Germany. The lack of familiarity with the term in Israel might also explain the stronger focus and longer discussions devoted to the current role of proxy and guardians in Israel compared with Germany. However, most of the German professional stakeholders also showed considerable unfamiliarity with the exact legal regulation and in regard to what ARD will mean in detail for future research practice.

When talking about implementation issues, both groups emphasized different areas. While professionals in Israel very intensively discussed the role of IRBs in the implementation of ARD and in monitoring the concrete research participation, professionals in Germany discussed the role of an IRB mainly in connection to risk–benefit assessment. Overall, there were different interpretations about the precise meaning of “minimal risk” or the exact definition of “potential benefit” for the research participant. The participants’ experiences varied in respect to how these two important criteria were currently interpreted and assessed in local IRBs. Dissent or even obscurity within both groups was expressed regarding whether, under the current law, studies with pure patient group benefit, but no or minimal risk (e.g., social scientific observational studies in care facilities or diagnostic studies based on blood examples) would be approved by different IRBs. Some participants reported they had never heard of any problems, while others reported that researchers had to go abroad for this kind of research because it is handled very strictly in their respective country. One German professional, as well as a patient organization representative, expressed skepticism toward ARD because the ARD practice already shows limitations regarding their interpretation.

**Figure 1:**
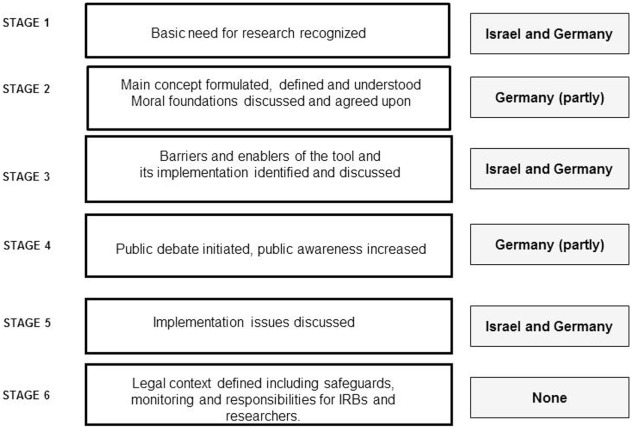
Suggested process of defining, regulating, and implementing ARDs and the observed situation in Germany and Israel.

Finally, Figure 1 presents an overview showing how far the process of discussing and implementing ARD in Germany and Israel has evolved. The figure indicates that while Germany is a bit more advanced regarding how to discuss the main concepts and involvement of the public, both countries are still in a rather preliminary stage of ARD implementation.

## Discussion

The present article compared professionals’ knowledge and attitudes regarding ARD in Israel and Germany. We identified a large spectrum of themes which were similarly discussed in both national focus groups, along with several differences. While the concept of ARD is relevant to other populations—such as psychiatric patients, ICU ventilated patients or patients with traumatic brain injuries, in both groups the focus was mainly on dementia, as a unique field for ARD implementation. Indeed, other populations were rarely mentioned by the participants in the current study because of two main reasons. First, the primary expertise of the participants in both countries was dementia. Second, while all these conditions are associated with difficulties to initiate ARD, unlike dementia, the other above-mentioned conditions do not have a specific, foreseeable trajectory and in some cases, chances of reversibility exist. Importantly, AHD is currently being intensively discussed for psychiatry (33), especially to enhance patients’ self-determination through potential phases of decision incapacity. However, as this review (33) reveals, the acceptance and uptake rate for AHD in psychiatry is still very low, and the question remains whether the willingness for research participation is not even lower.

Another important finding of our study is that there is a need to discuss the relevance and helpfulness of ARD in relation to different types of research. Research in dementia has tremendously evolved from single-patient studies from the Alois Alzheimer’s period to large-scale, international epidemiological or intervention studies with often more than thousands of patients. As the so-called “subsidiarity principle” (34) of the current EU directive (15) indicates, if the research in question can be answered by including competent patients, this should be the preferred research method. Individuals who lack competence should only be included in the study design if the research cannot be conducted without their inclusion. This subsidiarity principle for research with persons with dementia was already proposed in early work published on the topic of ARD in the 1990s (35). However, although much of the research about healthy aging will very likely include competent persons at first, longitudinal studies might have to deal with the loss of such competence—a point that can be addressed by the implementation of ARD ahead of time.

Another crucial topic addressed in both countries related to risk–benefit assessment and, more specifically, to the underlying definitions of risk and benefit. Regulations in Germany and Israel allow research with persons lacking competence only under the minimal risk/minimal burden paradigm (36). However, the question of what “minimal risk/minimal burden” actually entails in practice, is not always easy to answer (36). The latest version of the Declaration of Helsinki by the World Medical Association (37) addresses the topic of research with persons being unable to give informed consent in Article §24. Although the concept of ARD is not explicitly considered there, it is stated that informed consent should be obtained from the “legally authorized representative in accordance with applicable law”. In the U.S.A., according to the National Bioethics Advisory Commission, third-interest research with persons who are cognitively impaired is allowed under restrictions for proxy consent and minimal risk or if a legally authorized representative consents and there are no signs of objection by the incompetent person (38). The American College of Physicians (39) has added that if research participation entails more than minimal risk, a national IRB should review the research application.

In contrast, the German expert discourse on ethics and law is less permissive. For example, Germany has still not signed the “Oviedo Bioethics Convention” (40) developed by the Council of Europe in 1997. This is because the convention allows third-interest research with cognitively impaired persons. However, the Central Ethics Board of German Chamber of Physicians (41) suggested allowing such research, but only if there is a minimal burden, consent by a legal representative, and no opposing behavior on the part of the patient. In Israel, these topics are even less developed.

In sum, while ARD is an emerging concept internationally, a number of unsolved practical issues and ethical questions still await further clarifications. However, ARD remain an important tool for future research given their overall advantages. To advance knowledge in this area, it seems important that professionals from law, ethics and social sciences, as well as researchers in the field of healthy aging engage in a joint interdisciplinary and international discourse to exchange experiences regarding both the limits and benefits of such a tool, and to ensure best practice regarding information, monitoring and safeguarding mechanisms.

### Knowledge About ARD in the Public and Scientific Community

Our inspection of the literature and the knowledge emerging from the focus group study with professionals in the two countries showed that, while the importance of conducting research in the area of dementia and involving persons with dementia is increasing worldwide, the role and understanding of ARD to attain this goal is still in its developmental phases.

Conceptually, the definition of ARD is still blurred and the uniqueness of this tool compared with surrogate decision-making and other venues for anticipated decision-making, are not always clear. This theoretical fuzziness might explain the fact that public perceptions and knowledge about this tool is also lacking in research attention; the few studies that did examine these subject found very low prevalence rates. However, the low prevalence rates reported by these studies underline the importance of expanding knowledge in this area. Potential ways to attain this goal include: engaging the general public in a discourse on the topic *via* print and social media, and engaging with specific groups of affected persons, such as persons diagnosed with Mild Cognitive Impairment or early dementia *via* memory clinics or patient organizations. Indeed, a new project was recently initiated by the authors with the aim of elaborating in more detail on how to improve the public and scientific community’s knowledge and interests in ARD. In this new project (2018–2020), we will explore the extent to which patients and family members’ perspectives can actively contribute to a better conceptualization of ARD and related concepts, such as Advance Care Planning and communication about dementia, especially as prodromal and early diagnosis are undertaken more often.

In sum, while the legal status of ARD is still to be determined in each country regarding national laws and recommendations, it will gain relevance as more countries strive for legal and ethical harmonization in medical research, following the three main international documents dealing with the topic: the Council of Europe Convention on Biomedicine and Human Rights, its additional protocol on Biomedical Research, and the EU Directive 2001/20/EC on Clinical Trials on Medicine Products (15). Furthermore, if international cohort studies gain increasingly more relevance for healthy aging research, the interest in harmonization regarding research ethical standards might also increase, and ARD might serve as a promoter of this research.

### ARD as a Promoter of Healthy Aging Research

Similar to AHD and as discussed above, autonomy and self-determination are underlying principles of ARD (20). Thus, ARD might promote healthy aging in the area of dementia through two different although complementary avenues:

First, it might increase the amount of research conducted in the area by increasing the participation of persons with dementia who have consented *a priori* to be part of research projects. Second, it might facilitate researchers to conduct research in the field of dementia prevention if they know there is an available pool of persons who have completed ARD and might serve as potential participants. However, the first step in the process of executing ARD in order to improve the quality of life and death of persons with dementia should be providing knowledge and extending the awareness of professionals regarding the meaning and importance of this tool. Also the leading European patient organization, Alzheimer Europe (42) supports the use of advance directives for research. However, they mentioned that many practical and ethical issues regarding implementation, information and safe guarding are not yet sufficiently solved (pp. 59ff).

Another critical issue that remains is to clarify the meaning of “benefit” and for whom. For some, the distinction between patient group benefit and third-party benefit is too vague and even problematic. However, the current legal shift in Germany allowing research for the same “class of patient” resembles the existing U.S. guidelines (39). This additional dimension of benefit assessment needs additional normative justification and clarification. The first justification refers to the collective dimension. This is because historically any benefits related to the risk–benefit assessment in research ethics was addressed only to the individual patient-participant (43). The newer focus is now on other patients, rather than the patient-participant; this is based on the assumption that any clinical research should also have social value. To gain such social value, Buchanan and Miller (43) have suggested that any research design should explicitly address public health considerations. This would entail considerations that research should focus on treatments, cost effectiveness and fair access to such new treatments for a larger patient population. Research for healthy aging is likely to be in line with these social value conditions, but it is necessary to show this in a case-by-case manner. The second point refers to the conceptual and ethical issues: how and why to distinguish between “patients of the same class” and “other patients” when assessing collective benefit. Regarding patients’ altruistic motivations for research participation, for many it might be irrelevant whether only dementia patients would benefit from the research or only patients with another condition. The assumption that patients prefer to help patients within the same class of disease has—to our knowledge—not yet been empirically substantiated. ARD would be a chance to overcome this difficulty by giving citizens their own opportunity to set priorities.

### Limitations of Our Study

Comparing two countries such as Israel and Germany allows only limited representative knowledge regarding the professions on an international level. By covering various fields of expertise, we increased heterogeneity. The experts in our study were not randomly selected (which is always a difficult issue for expert interviews), but were identified by their professional backgrounds documented by their work profiles or academic CVs. Only two German experts and none of the Israeli experts had explicitly published on ARD, so for the most part, we had no particular ideas about what they would say during the focus group discussions. However, similar studies in additional countries, and including a wider variety of participants, will help provide a broader, more sustained picture.

### Summary and Conclusion

Implementing a new research or organizational tool is a step-by-step procedure requiring a thorough understanding of the current state of knowledge, as well as the challenges and hurdles ahead. Thus, this article aimed to describe the state of knowledge in the area of ARD and to discuss the main ethical and practical dilemmas in their implementation, while comparing Israeli and German professional stakeholders’ perspectives on the topic.

Overall, from our qualitative exploration of focus group discussions, several similarities and dissimilarities between the countries emerged. While differences in the cultural and legal environments of both countries might explain these finding, they may also reflect the fact that these societies are in different stages of the ARD implementation process. First, as represented in Figure 1, a complete analysis of the focus groups showed that in both countries the evolution of ARD seems to follow a process similar to the development and implementation of new medical technologies. While both countries are in the midst of a developmental process and have recognized the importance and the need for ARD as a tool for expanding healthy aging research, Germany is in a more advanced stage than Israel. This is because of the EU regulation process, which indicates the influence of international harmonization on these research ethical issues.

As long as improving quality of life and promoting autonomy continue to be core elements in the process of healthy aging, efforts to advance knowledge and solve dilemmas associated with ARD implementation is of the utmost importance. This article provided a small but important step in this direction.

## Ethics Statement

The University of Haifa Ethics Committee approved this project. In Germany, no IRB is needed for collecting data by interviewing experts or professionals’ perspectives. However, all participants, in Germany as well as in Israel received an information sheet and a consent letter explaining the aim of the study and ensuring their consent for a pseudonymonized data analysis and publication of data in a fully anonymized way.

## Author Contributions

PW and SiS conceptualized the questionnaire for the focus groups and jointly analyzed the material and wrote the article. SiS facilitated both focus groups as the moderator and conducted the additional expert interviews.

## Conflict of Interest Statement

The authors declare that the research was conducted in the absence of any commercial or financial relationships that could be construed as a potential conflict of interest.
