# Supplementary material for: Practical and Ethical Aspects of Advance Research Directives for Research on Healthy Aging: German and Israeli Professionals’ Perspectives
Source: Front Med (Lausanne). 2018 Apr 5;5:81. doi: 10.3389/fmed.2018.00081 (PMC5895655; doi:10.3389/fmed.2018.00081)
Supplement: Supplementary file 1 [file data_sheet_1.PDF]

Silke Schicktanz, Perla Werner, Ile Kermel Schiffmann (Draft 19th Feb 2017)

Expert focus groups discussions on Advance Research Directives

Open Questionnaire:

I. Intro: Experience and background knowledge

(1) Let us begin by introducing briefly your expert background. Please say very briefly why you are interested in taking part in this focus group.

(2) Have you already heard about Advance Research Directives[1]? In what context?

In the USA, NL and Germany, ARD are now discussed as an appropriate tool, especially for dementia research because proxy consent is not always seen as a sufficient legal or ethical solution to ensure consent.

(3) What do you think of the idea of an ARD? Do you think it will facilitate or rather hinder research and innovation in dementia, and if so, for what reasons? Do you have also other illness in mind, for which ARD can be meaningful?

II. Content and practice of ARD

(4) Consider the following case: Person A has designed and agreed in an ARD that she is willing to take part in pharmaceutical research even if she would not benefit herself from it and if there would be likely also severe side-effects such as severe nausea or a kidney failure:

- Would be such an ARD still valid when the person is then in a late stage of dementia?
- Under which conditions would be such an ARD invalid according to your moral attitudes (e.g. proxy/relative does not agree, burden of risk is too high, signs of discomfort of the patient)
- (Do have proxies or relatives always a right to vote against an ARD?)
- Is there any research (regarding specific aims or methods) which is particularly challenging for ARD (e.g. pharmaceutical, diagnostics, preventive, third party)? Why? Where do you see the main challenges in dementia research?

[1] In the questionnaire we use the abbreviation ARD, but it will always addressed during the FGs as full term

(5) How detailed could and should an ARD be composed to be useful in practice? Is there any particular information for the public needed or already available to empower them to compose and sign an ARD?

(6) When would be a good time to ask persons to write an ARD and if so, who should be encouraged to write an ARD?

(7) Do you see any particular ethical or legal challenges to the implementation of ARD?

IV: Advanced planning of health care issues

(8) Which parallels regarding the ethical, social, legal or religious issues do you see between Advanced Research Directives and Advance Care Directives?

(9) Actually, the number of people in the general public but also even in particular patient groups holding an AD in Israel is very low (as in many other industrialized countries): What should be done according to your opinion to overcome public hesitance towards AD? Which needs have to be addressed to encourage or make people willing to sign ADs?

V. (if not addressed yet) Dementia Research Setting as such

(10) How is currently research participation of person with dementia regulated in Israel? How is consent reached, and what role do proxies, relatives or power of attorneys play?

V. Open end

(11) Is there anything else, we have yet not talked about, but you feel it is necessary for discussing the topic?
